# Respiratory Syncytial Virus Infection Induces Higher Toll-Like Receptor-3 Expression and TNF-α Production Than Human Metapneumovirus Infection

**DOI:** 10.1371/journal.pone.0073488

**Published:** 2013-09-09

**Authors:** Ying Dou, Yao Zhao, Zhi-yong Zhang, Hua-wei Mao, Wen-wei Tu, Xiao-dong Zhao

**Affiliations:** 1 Laboratory Biosafety-2, Institute of Pediatrics, Children’s Hospital of Chongqing Medical University, Chongqing, China; 2 Ministry of Education Key Laboratory of Child Development and Disorders, Children’s Hospital of Chongqing Medical University, Chongqing, China; 3 Key Laboratory of Pediatrics in Chongqing, Children’s Hospital of Chongqing Medical University, Chongqing, China; 4 Chongqing International Science and Technology Cooperation Center for Child Development and Disorders, Children’s Hospital of Chongqing Medical University, Chongqing, China; 5 Department of Pediatrics and Adolescent Medicine, The University of Hong Kong, Hong Kong, China; University of Georgia, United States of America

## Abstract

Respiratory syncytial virus (RSV) and human metapneumovirus (hMPV) are common causes of respiratory infections in children. Diseases caused by hMPV are generally considered to be less severe than those caused by RSV; the underlying mechanisms, however, remain unknown. In the present study, the expressions of TLRs in airway epithelial cells and lungs of BALB/c mice infected by hMPV or RSV were measured in an attempt to explore the differences in the airway inflammation caused by the two viruses. Our results demonstrate that both hMPV and RSV infection upregulated the expressions of TLRs and inflammatory cytokines. Specifically, the TLR3 expression was revealed to be elevated *in vitro* and in mouse lungs. IFN-α produced by A549 cells after RSV or hMPV infection remained undistinguishable, whereas production of TNF-α was significantly higher after RSV infection than hMPV infection either in the presence or absence of Poly I:C. This study provides a clue that more severe clinical syndrome of RSV infection may be due to the greater magnitude of induction of airway inflammation by RSV involving TLR3 activation and production of TNF-α.

## Introduction

Respiratory syncytial virus (RSV) is a nonsegmented, single-stranded, enveloped, negative-strand RNA virus that belongs to the *Paramyxoviridae*, *Pneumovirus* genus. RSV is the leading viral pathogen that causes lower respiratory infections in infants and young children worldwide [1]. After RSV infection, the airway epithelial cells produce many immunologically active molecules, such as cytokines, chemokines and reactive oxygen species, which promote the occurrence of bronchiolitis and pneumonia in infants and young children [2]. Human metapneumovirus (hMPV), which was first isolated in 2001, is a new member of *Pneumovirinae*, *Paramyxoviridae* and causes respiratory infections in all age groups, even death in immunodeficienct patients [3,4]. HMPV is genetically similar to RSV [5,6], and the clinical sympotoms of hMPV and RSV infections are generally indistinguishable. Both of them can cause bronchitis, bronchiolitis or pneumonia in infants and young children [7]. Although the spectrum of respiratory disorders associated with the two viruses are similar, the disease severity caused by hMPV is milder than that by RSV [8,9,10]. For instance, the duration of symptoms and treatment time were shorter in hMPV-infected patients, and more RSV-infected children required respiratory support [8]. The underlying mechanism of the different disease severity is currently unknown.

As the first line of host defense strategy, the innate immunity plays a critical role in antiviral immune responses by direct combating against the pathogens and shaping the development of adaptive immunity. Toll-like receptors (TLRs) are important components of the innate immunity. Upon pathogen associated molecular pattern (PAMP) ligation, TLRs activate innate immune cells and regulate immune responses [11,12]. One of the most fundamental TLR-mediated effects is the activation of NF-κB signaling and subsequent regulation of the production of proinflammatory cytokines and chemokines, which are strongly associated with the outcome of inflammatory disease. RSV F protein can be recognized by TLR4 [13], numerous studies have also demonstrated TLRs can recognize other components of RSV, including dsRNA (TLR3) and ssRNA (TLR7/8) [13,14,15]. The activation of these TLRs on RSV-infected airway epithelial cells results in the secretion of cytokines and chemokines including type I interferons (IFN) and tumor necrosis factor alpha (TNF-α) that play a critical role in regulating local inflammatory processes in the lung and subsequent tissue damage. Interferons (IFNs) are a heterogeneous family of cytokines with demonstrated antiviral, antitumor, and immunomodulatory activities. Local production of IFN-α/β plays an important defensive role in many respiratory virus infections by limiting viral replication until virus-specific host defense mechanisms develop [16,17]. And tumor necrosis factor alpha (TNF-α) is important proinflammatory cytokines, prior studies suggest that TNF-α plays an important role in the process of pulmonary inflammation caused by RSV infection [18,19]. It is generated by the regulation of nuclear factor kappa B, the activation of NF-κB can produce large amounts of TNF-α cause immune and inflammatory response [20]. TNF-α was shown to associate with neutrophil activation and migration to the lungs, while in RSV infection, neutrophils account for more than 90% of the inflammatory cells present in the upper respiratory tract and tracheal fluid [19,21,22]. In addition, TNF-α can also induce lung injury through direct cytotoxic effects on alveolar epithelial cells [23].

HMPV is also a member of the Paramyxoviridae family, but the correlation between TLR activation and hMPV infections is still unclear. In the present study, we proposed to explore whether RSV and hMPV infection induce distinct profiles of TLR activation and inflammatory response, aiming to clarify the mechanisms underlying the different disease severity caused by the two viruses.

Epithelial cells in the respiratory tract are both prime targets of respiratory viruses and the earliest source of innate antiviral immunity; thus, these cells play a critical role in regulating the respiratory tract inflammation that occurs upon viral infection [24]. Therefore, in this study, A549 cells (a human lung adenocarcinoma epithelial cell line) and airway epithelial cells of BALB/c mice were infected with hMPV or RSV, and the expression of TLRs and their roles in infection were investigated *in vitro* and *in vivo*. Compared to hMPV, RSV induced higher TLR3 expression and TNF-α production, which may contribute to the more severe airway inflammation and lung tissue injury.

## Methods

### Materials

The following products were used in this study: fetal bovine serum (FBS) and DMEM medium (GIBCO, USA), RPMI1640 (HyClone, USA), EZgeno total RNA extraction kit (Genemega, USA), PrimeScript RT kit (Takara, Japan), 2×Taq PCR MasterMix and RealMasterMix (SYBR Green; Beijing Tiangen Biotech, China), phycoerythrin (PE)-labeled monoclonal IgG1α hTLR3 antibody and PE-labeled mIgG1 isotype control (eBioscience, San Diego, CA), Poly(I:C) (InvivoGen, USA), and human IFN-α, TNF-α and IL-8 ELISA kits (Bender MedSystems, Austria).

### Preparations of hMPV and RSV

Recombinant hMPV NL/1/00 was prepared using the reverse genetics method (Briefly full- length cDNA clones of hMPV NL/1/00 and 4 helper vectors (pCITE-N, pCITE-L, pCITE-P and pCITE-M2.1 for expressions of major viral proteins as a gift from Holland professor Erasmus Institute Fouchier were co-transfected into BSR-T7 cells to rescue live virus by using reverse genetics technique) was cultured on Vero-E6 cells in DMEM medium containing 3% FBS, 2 mM L-glutamine, 0.25 mg/mL trypsin, 100 U/mL penicillin and 100 µg/mL streptomycin in an incubator with 5% CO_2_ at 37^°^C. RSV A2 (obtained from the Viral Laboratory in Beijing Children’s Hospital) was grown on Hep-2 cells in RPMI1640 medium containing 2% FBS, 100 U/mL penicillin and 100 µg/mL streptomycin in an incubator with 5% CO_2_ at 37^°^C. The infected cells and culture medium were freeze-thawed when the CPE reached 80%. Both hMPV and RSV were purified by sucrose gradient centrifugation as described elsewhere [25,26]. Virus titers were determined by a plaque assay and were expressed as PFUs/ml. The purified hMPV and RSV were aliquoted and stored at -80°C for further experiment. Ultraviolet (UV) light-inactivated RSV and hMPV were prepared as previously described [27].

### Culture and viral infection of epithelial cells

A549 cells were purchased from the American Type Culture Collection and maintained in DMEM containing 10% FBS, 2 mM L-glutamine, 100 U/mL penicillin and 100 µg/mL streptomycin. These cells were seeded in 6-well plates at 3×10^5^ cells/well and inoculated with hMPV, RSV, UV-hMPV or UV–RSV when the confluence reached ~80%. The virus maintenance medium was refreshed 1 h later, followed by incubation in an incubator with 5% CO_2_ at 37^°^C for varying durations.

### Growth kinetics in vitro

A549 cells were inoculated with hMPV and RSV at a multiplicity of infection (MOI) of 0.1, and 1 ml of medium from each well was harvested at 24 h intervals for up to 6 days post-infection. A plaque assay was performed to determine the viral titers in PFU per milliliter.

### Detection of TLR mRNA levels in A549 cells using RT-PCR and real-time quantitative PCR

Cells were harvested at 3, 6, 9, 12 and 24 h after virus infection at an MOI of 2, and the total RNA was extracted using the EZgeno total RNA extraction kit according to the manufacturer’s instructions. cDNA was synthesized by reverse transcription according to the instructions of the PrimeScript RT kit. A total of 2 µl of cDNA was PCR amplified in a 25 µl reaction mixture at an annealing temperature of 55-65 ^°^C for 35 cycles. The PCR products were separated by 1.5% agarose electrophoresis and scanned with a gel imaging system. Then the electrophoresis strips were analyzed by Quantity One software. The mRNA levels of TLR1-10 were semi-quantitatively determined and normalized to the expression of β-actin. RT-PCR results were used for screening.

The SYBR Green I fluorochrome method was employed to perform real-time quantitative PCR with the cDNA products synthesized as described above. The 25 µl-reaction mixture included 1 µl of cDNA, 11.25 µl of RealMasterMix SYBR Green and 0.5 µl of each primer. The reaction was conducted in a CFX96 Fluorescence Thermocycler (Bio-Rad) under the following conditions: pre-denaturation at 95°C for 3 min followed by 45 cycles of denaturation at 95°C for 10 sec, annealing at 60°C for 10 sec, and extension at 68°C for 10 sec. The experiments were performed in triplicates, and melting curve analysis was performed to confirm that no nonspecific amplification occurred. The Ct value of each sample was obtained, and the relative expression of target genes was calculated based on the difference between the Ct values of the target genes and housekeeping genes, which is represented as 2^△△Ct^. The primers for TLR1-10 were modified from Sha et al. [28]. The primers used for the β-actin amplifications are as follows:

Forward: 5’-AAGATGACCCAGATCATGTTTGAGACC-3’,

Reverse: 5’-AGCCAGTCCAGACGCAGGAT -3’.

### Animals and virus inoculation

All animal work was approved by The Institutional Review Board of Chongqing Medical University. Six- to eight-week-old female pathogen-free BALB/c mice were purchased from and bred at the specific pathogen-free (SPF) Animal Center of Chongqing Medical University. Experiments were conducted in a Biosafety-2 laboratory at the Children’s Hospital of Chongqing Medical University. A total of 144 mice were randomized into three groups: including the hMPV group, the RSV group and the control group (n=48 per group). After anesthetization, the mice in the hMPV and RSV groups were infected intranasally with 1.5×10^6^ PFU hMPV NL/1/00 or RSV A2, respectively, while the mice in the control group were inoculated intranasally with 100 µl of DMEM. The animals were anesthetized and sacrificed at days 1, 3, 5, 7, 9 and 16 after inoculation.

### Viral replication kinetics in mouse lungs and histopathology

The lungs of the infected mice were harvested at various times after inoculation. The right lungs were weighed and homogenized on ice in 10-fold volumes of HBSS containing 0.218 M sucrose, 25 mM HEPES, 4.8 mM L-glutamate, 200 U/ml penicillin and 200 µg/ml streptomycin (pH 7.4). A plaque assay was performed with the homogenates to determine the viral titer. The resulting titer for each sample was divided by the lung weight and reported as PFU per gram of lung. The left lungs were fixed with 4% paraform for histopathology. The fixed lungs were embedded in paraffin, sectioned into 4 µm slices, and stained with hematoxylin-eosin. The histopathologic score (HPS) was determined using the method described by Cimolai [29]. A final score per animal (ranging from 0 to 26) was obtained by averaging scores from each lung which had been accumulated by the addition of subscores from the assessments of quantity and quality of peribronchiolar and peribronchial infiltrates, luminal exudates, perivascular infiltrates, and parenchymal pneumonia.

### Detection of mRNA expression of TLR3, TLR4, TLR7 and TLR8 in the mouse lungs by real-time quantitative PCR

The lungs were homogenized and total RNA was extracted according to the instruction of EZgeno total RNA extraction kit. Reverse transcription was performed to synthesize cDNA according to the instructions of the PrimeScript RT kit. The SYBR Green I fluorochrome method was used for real-time quantitative PCR following the same procedures described above. Primers specific for mouse TLR3-4, TLR7-8 and β-actin were used as previously described [30].

### Detection of TLR3 protein expression in A549 cells by flow cytometry

After pre-treated with Poly(I:C) (10 µg/mL) for 6 h, A549 cells were infected with hMPV or RSV at an MOI of 2 at 37°C for 24h. Cells were harvested and labeled with either the phycoerythrin (PE)-labeled anti-human TLR3 monoclonal antibody (clone TLR3.7) or PE-labeled mIgG1 isotype control. For intracellular staining, cells were fixed with a solution of 3% paraformaldehyde in phosphate buffered saline (PBS) for 10 min on ice and permeabilized by the addition of 0.2% Tween-20 in PBS for 15 min at 37°C, and then stained with anti-hTLR3 for 30min. The cells used for surface staining were not fixed. Fluorescence analysis was performed using a FACS Calibur machine with the FACScan software. Ten thousand live cells were counted, and viability was determined by generating forward scatter versus side scatter density plots and setting the gate to exclude dead cells. Histogram plots included only gated cells.

### Measurement of IFN-α, TNF-α and IL-8 levels by ELISA

A549 cells were seeded in 6-well plates at 3×10^5^ cells/well. When the cells reached ~80% confluence, the culture medium was refreshed with serum-free DMEM. Cells were pre-treated with Poly(I:C) (10 µg/mL) for 6 h followed by infection with hMPV or RSV at a MOI of 2 at 37^°^C for 24 h. The supernatants were collected and centrifuged at 14000 r/min for 10 min. IFN-α, TNF-α and IL-8 levels were determined with ELISA kits (Bender MedSystems) according to the manufacturer’s instructions. The concentration of the IFN-α standards ranged from 7.8 to 500 pg/ml, the TNF-α standards ranged from 23 to 1500 pg/ml and the IL-8 standards ranged from 15.6 to 1000 pg/ml.

### Statistical analysis

Statistical analyses were performed using the SPSS version 11.0 statistics software, and the data are expressed as the means ± standard deviation (SD). The results from the plaque assay (viral titers), RT-PCR and histopathologic experiments were analyzed using paired *t*-tests, those from real-time quantitative PCR and flow cytometry measurements were analyzed using the Kruskal-Wallis test, and data from the ELISAs were analyzed using a one-way analysis of variance (ANOVA) test. A value of P<0.05 was considered to be statistically significant.

## Results

### Assessment of growth kinetics of hMPV and RSV in A549 cells and in the lungs of infected mice

**Figure 1:**
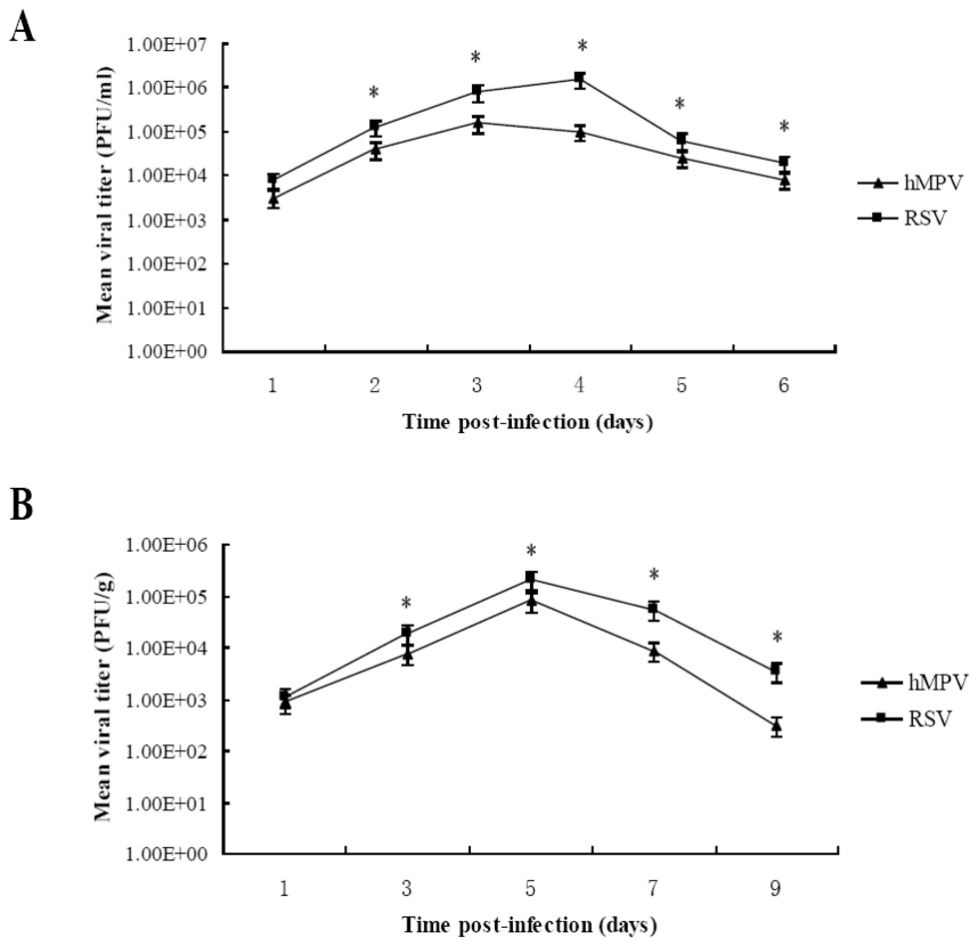
Growth kinetics of hMPV and RSV in A549 cells and mouse lungs. A) A549 cells were infected with RSV or hMPV at an MOI of 0.1. At the indicated time points the virus-containing media were collected and determined for the viral titer by plaque assay. Viral titer was expressed as log PFU/ml culture media. * *P*<0.05 vs. hMPV titer on the corresponding time points. B) Eight mice in each group were infected with 1.5×10^6^ PFU hMPV or RSV. At the indicated time points post infection, the mice were sacrificed. Mouse lungs were homogenated and then subjected to viral titration by the plaque assay on Vero E6 cells and Hep-2 cells. Viral titer was expressed as log PFU/g lung tissue. * *P*<0.05 vs. hMPV titer on the corresponding time points.

First of all, in order to know the replication of the two viruses, we assessed the growth kinetics. The growth kinetics of hMPV and RSV were compared in A549 cells and the lungs of infected mice. The hMPV titers peaked at approximately 10^5^ PFU/ml in the A549 cell cultures on the third day after inoculation, whereas the RSV titers peaked at 10^6^ PFU/ml in the cultures on the fourth day after inoculation (Figure 1A). Viral titers in the lungs of hMPV- infected BALB/c mice peaked on the 5th day post-infection [(8.16 ± 0.78) ×10^4^ PFU/g], and remained at detectable levels until the 9th day post-infection [(3.08 ± 1.02) ×10^2^ PFU/g]. Titers in the lungs of RSV-infected BALB/c mice also peaked on the 5th day post-infection at a level of (2.12±0.67) × 10^5^ PFU/g, which was higher than the titer in lungs from hMPV-infected mice (Figure 1B).

### Lung histopathology after infection with hMPV or RSV

**Figure 2:**
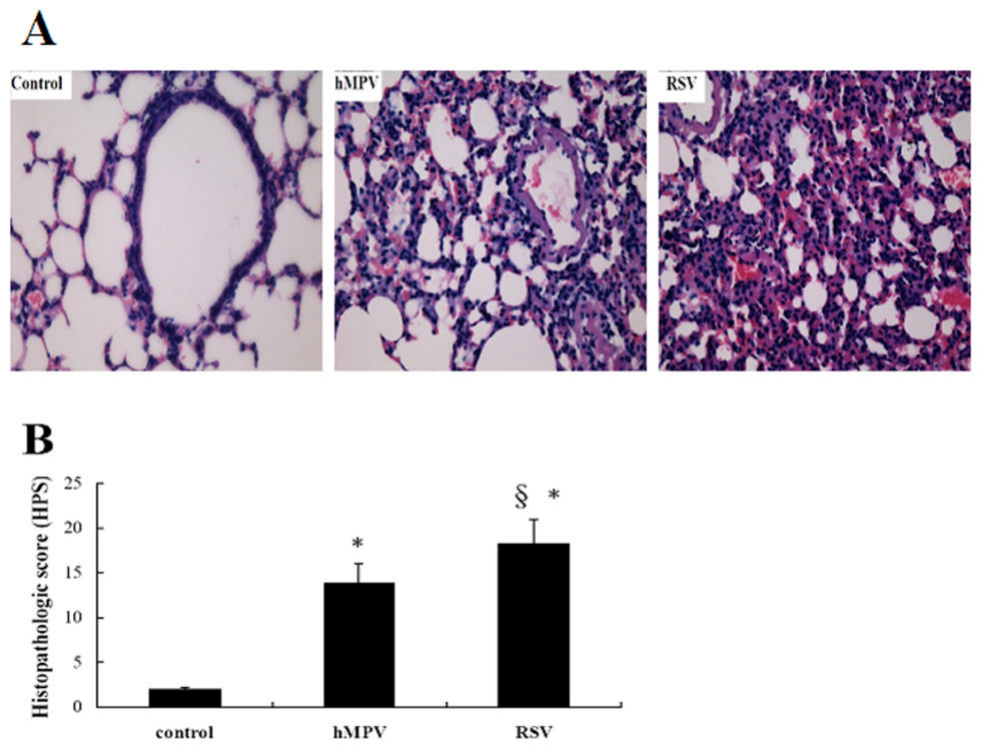
Lung histopathology of infected mice on day 5 after hMPV or RSV infection. Eight mice in each group were sacrificed on day 5 post-infection and lungs were harvested, fixed with 4% paraform, embedded in paraffin, cut into sections and stained with haematoxylin-eosin (H and E). A) Representative lung section, magnification×200. B) The histopathologic score (HPS) was determined and data were expressed as mean ±SD (n=6). * *P*<0.05 vs. control, ^§^ *P*<0.05 vs. hMPV-infected mice.

Through the growth kinetics, we found the RSV titers were higher than that of hMPV in mice, then we tested the lung histopathology. The lungs from the control group mice had clear alveoli without inflammatory infiltrates around bronchioles or vessels and a low histopathologic score (HPS) of 1.96±0.42 (n=8). A clear inflammatory response was observed on the 5th day post-infection in both hMPV- and RSV-infected mice and was characterized by swelling of bronchiolar epithelia, alveolar dilation and extensive infiltration that was dominated by an excess of lymphocytes and macrophages surrounding both bronchioles and pulmonary blood vessels (Figure 2A). RSV-infected mice had a higher mean HPS than that of hMPV-infected mice (Figure 2B), indicating that RSV-induced airway inflammation was more severe compared with that induced by hMPV.

### Messenger RNA expression levels of TLRs in A549 cells infected with hMPV or RSV

**Figure 3:**
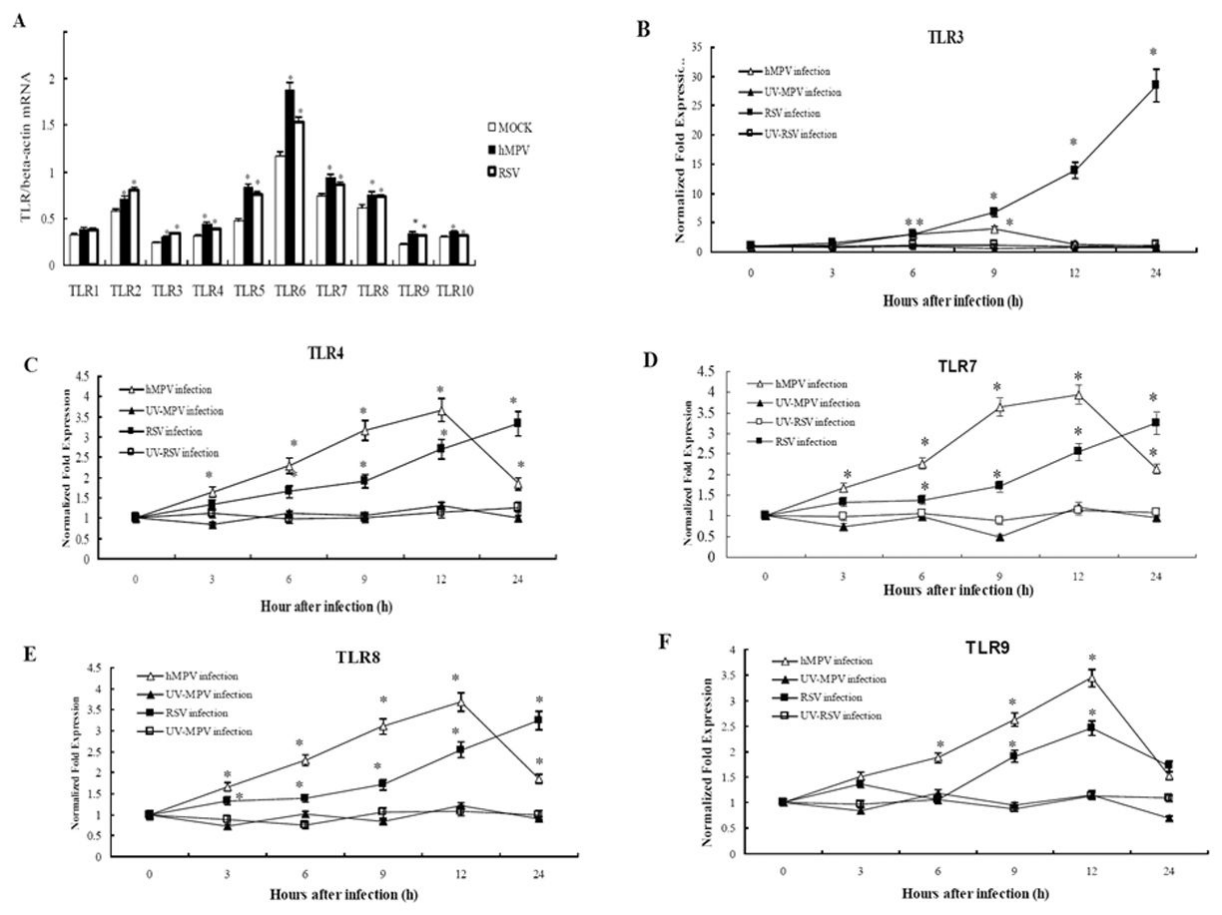
The mRNA expressions of TLRs in A549 cells after hMPV or RSV infection. A549 cells were inoculated by hMPV, RSV, UV-MPV or UV–RSV at an MOI of 2, and cells were harvested at 3, 6, 9, 12 and 24 h after infection for total RNA extraction**. A**) The TLR1-10 mRNA levels were detected by RT-PCR at 6 h after hMPV or RSV infection. Values of TLR1-10 mRNA/beta-actin mRNA were expressed as mean ±SD (n=6). Normal A549 cell served as the MOCK. * *P*<0.05 vs. MOCK. **B**) **to F)** were the dynamic expression of TLR3-4, TLR7-9 respectively. The TLR3-4 and TLR7-9 mRNA levels were detected by real time RT-PCR at 3, 6, 9, 12 and 24 h after inoculated by hMPV, RSV, UV-MPV or UV–RSV. β-actin served as an internal reference, and untreated cells as control. Data were expressed as mean ±SD (n=6). * *P*<0.05 vs control.

To further examine possible causes of airway inflammation, we screened the TLR expression profile in RSV- or hMPV-infected A549 cells by RT-PCR. As shown in Figure 3A, at steady state, A549 cells did express some amount of TLRs, but the levels of most TLRs were up-regulated after hMPV or RSV infection. Compared with the control cells, the levels of TLR2-10 increased to different extents (*P*<0.05) at 6 h after hMPV infection, while the levels of TLR2-8 were all significantly elevated 6 h after RSV infection (*P*<0.05) (Figure 3A). However, A549 cells treated by UV-hMPV or UV–RSV did not exhibit significant changes in TLR levels (data not shown). Previous studies show that TLR3-4 and TLR7-9 play an important role in RSV infection. So we further detected the levels of those TLRs using real-time quantitative PCR. As shown in Figure 3B–F, the levels of TLR3-4 and TLR7-9 were increased 3 h after hMPV infection. The TLR3 levels peaked at 9 h after hMPV infection, and the levels of TLR4 and TLR7-9 peaked at 12 h after infection, then started to decline. In contrast, during 24 hours of RSV infection, the levels of TLR3-4 and TLR7-8 were increased from 3 h after RSV infection, and reached to the highest levels at 24 h after infection, whereas TLR9 expression reached a peak level at 12 h after infection with RSV, and then declined. Importantly, the increase of TLR3 expression induced by RSV was the highest among all the TLRs measured here. In addition, the increase of TLR3 induced by RSV was also significantly greater than that induced by hMPV. The treatment with UV-hMPV or UV–RSV did not change the expressions of TLR3-4 and TLR7-9 in A549 cells (Figure 3B–F).

### TLR expression levels in the lungs of mice infected with hMPV or RSV

**Figure 4:**
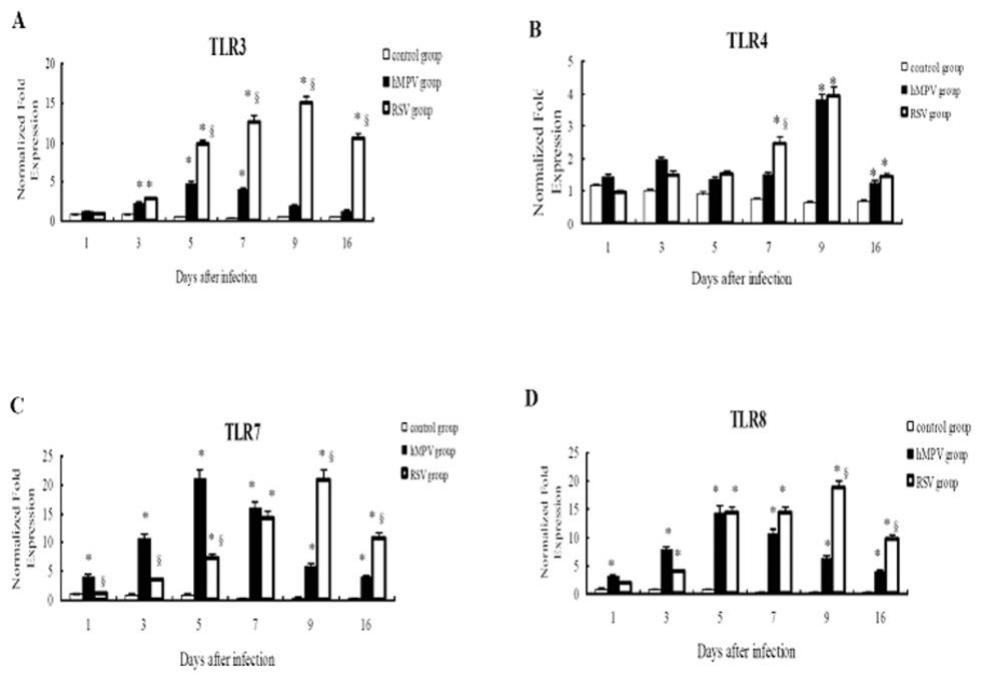
Messenger RNA expressions of TLRs in the lungs of BALB/c mice after hMPV or RSV infection. Mice in HMPV group, RSV group and control group were sacrificed at 1, 3, 5, 7, 9 and 16 days after infection, and the lungs were harvested for extraction of total RNA and determination of mRNA expressions of TLR3-4 and TLR7-8 by real-time PCR. β-actin served as an internal reference. Data were expressed as mean ±SD (n=8). * *P*<0.05 vs. control; ^§^ *P*<0.05 vs. hMPV group. A) to D) were the expression of TLR3-4, TLR7-8 respectively.

In order to know whether the change of TLRs expression in vitro was consistent with that in vivo model, we further investigated part of the TLRs in the lungs of BALB/c mice after hMPV and RSV infection. The levels of TLR3-4 and TLR7-8 were up-regulated in the lungs of hMPV-infected mice. The levels of TLR3 and TLR7-8 peaked at 5 days after infection, while the levels of TLR4 peaked at 9 days after infection. After RSV infection, the levels of TLR3-4 and TLR7-8 in the lungs were also significantly up-regulated, and the levels of all four TLRs peaked at 9 days after infection. Importantly, the TLR3 levels in the RSV-infected lungs were significantly higher than those in the hMPV-infected lungs (Figure 4A–D). These results are consistent with those from the *in vitro* experiments. Furthermore, the levels of TLR7-8 were markedly higher than that of TLR3-4 in hMPV-infected group, and also markedly higher than TLR4 in RSV group. This is different from the *in vitro* experiments. Whether TLR7-8 pathway play an more important role in mice than in human body was still unclear.

### Expression of TLR3 protein in A549 cells infected by hMPV or RSV

**Figure 5:**
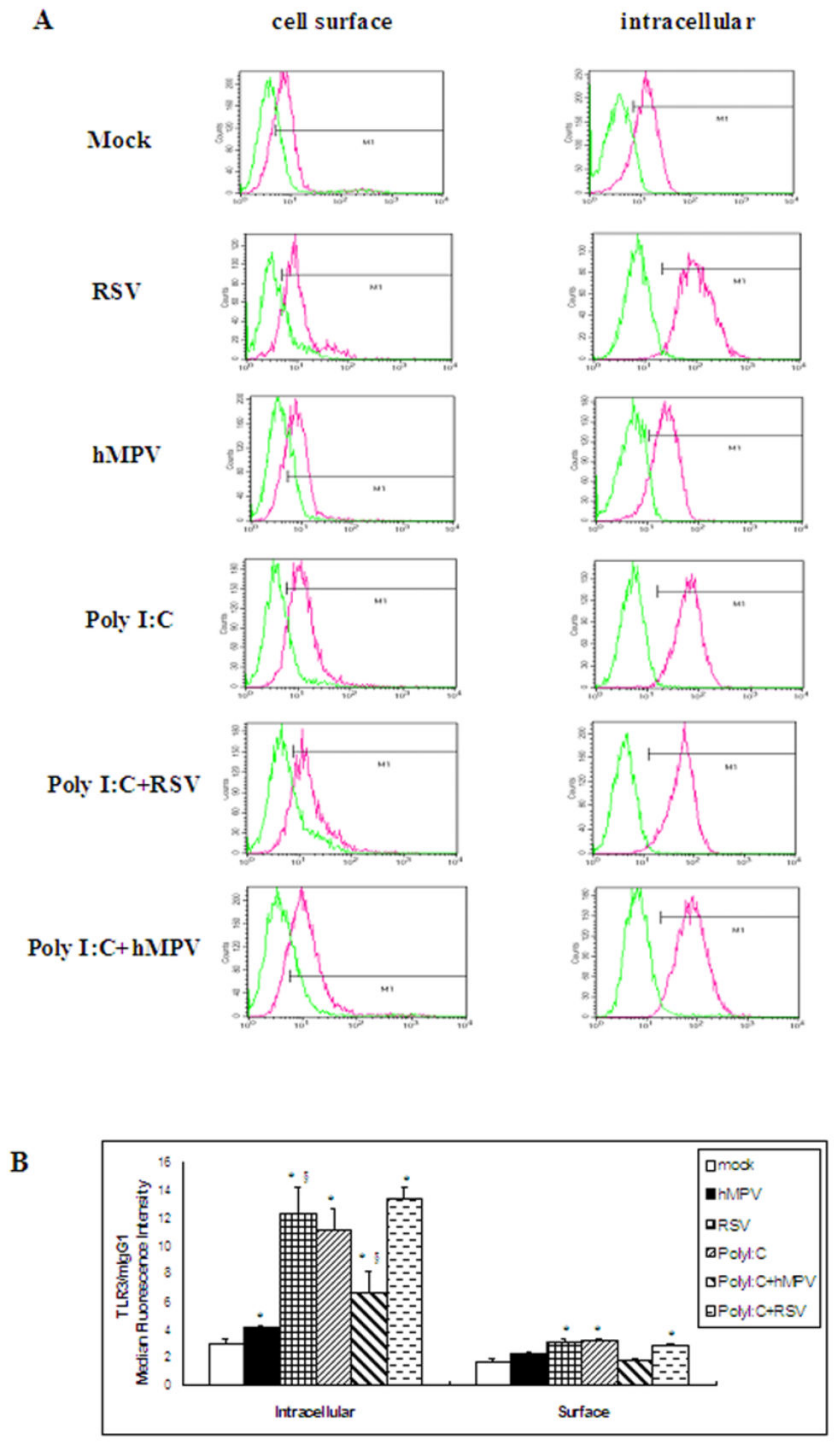
Expression of TLR3 protein intracellular or on the surface of A549 cells by flow cytometry. A549 cells were pre-treated with Poly(I:C) (10 µg/mL) for 6 h, then infected by hMPV or RSV at an MOI of 2 at 37°C for 24h. Cells were harvested for assessing TLR3 expression by flow cytometry. Cell surface and intracellular staining were shown. TLR3 protein was located mainly intracellularly. A) Red histograms represent staining with the specific antibody underlaid with the isotype-matched control antibody (green histograms). B) Data were expressed as mean ±SD (n=6). * *P*<0.05 vs. mock; ^§^ *P*<0.05 vs. hMPV.

The results of real-time quantitative PCR indicated the TLR3 mRNA were significantly increased after RSV infection compared with that after hMPV infection both *in vitro* and *in vivo* experiments. In order to know whether the expression of TLR3 protein was consistent with the expression of mRNA, we detected the expression of TLR3 protein in A549 cells after hMPV or RSV infection. Uninfected A549 cells express low intracellular and almost no surface TLR3 protein. Cell surface expression of TLR3 compared with the isotype control was detected in only 1% of cells, and intracellular TLR3 was detected in 3% of cells (Figure 5A). After infection with RSV or treatment with PolyI:C, the expression of intracellular TLR3 significantly increased. hMPV infection also induced an increase in the intracellular TLR3 levels, but the increase was significantly lower than that induced by RSV or PolyI:C. However, the levels of intracellular TLR3 in the hMPV-PolyI:C group were significantly higher than those in the hMPV alone group (Figure 5A,B).

### Secretion of IFN-α, TNF-α and IL-8 by A549 cells infected with hMPV or RSV

**Figure 6:**
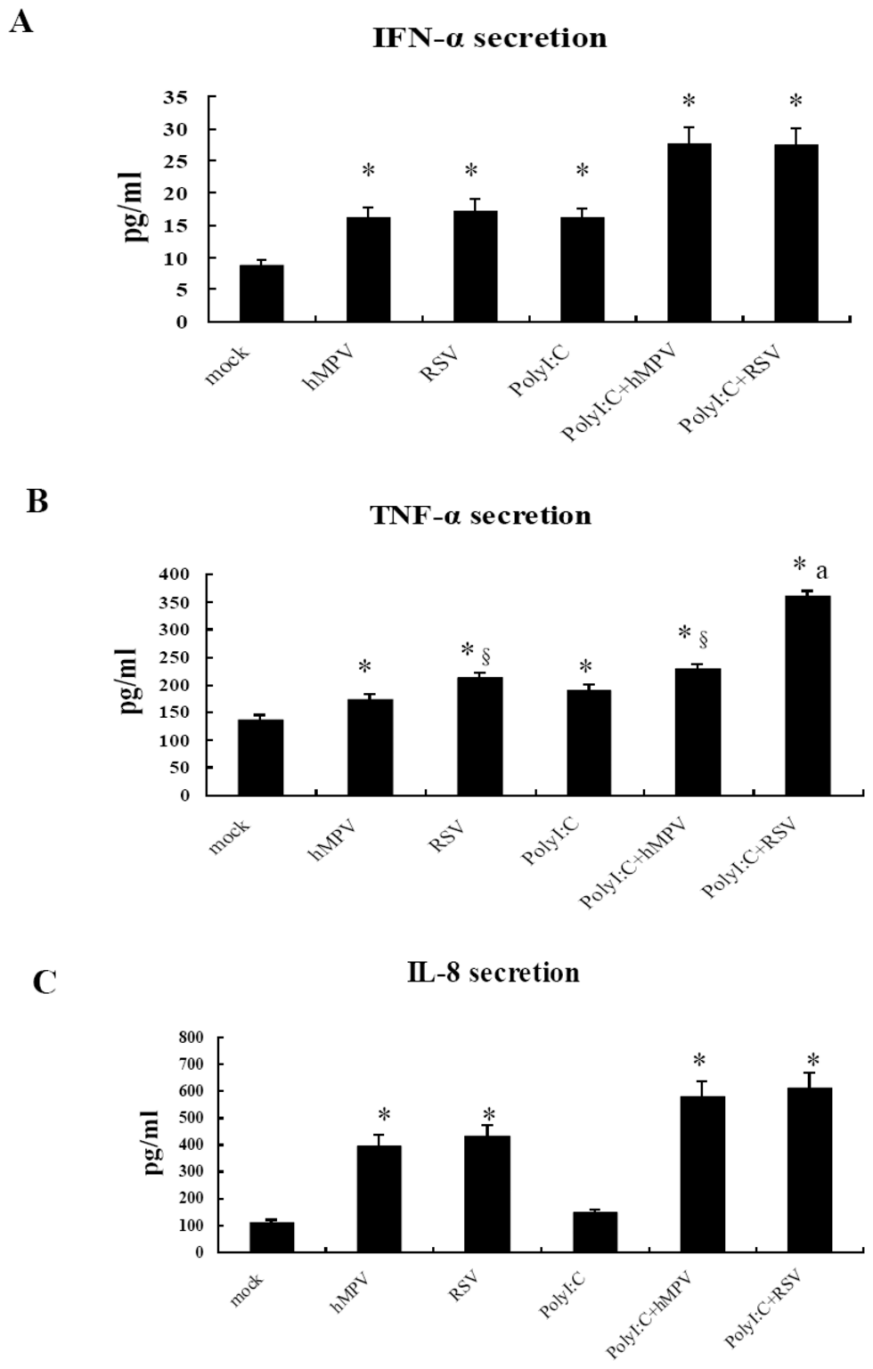
Secretion of IFN-α, TNF-α and IL-8 from A549 cells after infection of RSV or hMPV or pretreated with PolyI:C. A549 cells were seeded in a 6-well plate, and culture medium was replaced with serum free DMEM on the second day. Some were pretreated with Poly(I:C) (10µg/mL) for 6 h, and then infected by hMPV and RSV at an MOI of 2 at 37°C for 24 h. The supernatants were collected and ELISA was performed to detect the production of IFN-α, TNF-α and IL-8. Data were expressed as mean ±SD (n=6). * *P*<0.05 vs. normal control; ^§^ *P*<0.05 vs. hMPV; ^a^ *P*<0.05 vs. PolyI:C+hMPV.

Previous studies have suggested that the activation of TLRs can induces expressions of proinflammatory cytokines and chemokines, which are strongly associated with the outcome of inflammatory disease, via the IRF-3 and Nf-κB pathways or TRIF and TRAM pathways [31]. To study the expressions of proinflammatory cytokines and chemokines after hMPV and RSV infection, we chose IFN-α, TNF-α and IL-8 as examples. Uninfected A549 cells have relatively low levels of IFN-α. However, after infection with hMPV or RSV or treatment with PolyI:C, the levels of IFN-α significantly increased above the baseline level, with no significant difference between the hMPV- and RSV-infected cells. The IFN-α levels were significantly higher in the hMPV-PolyI:C and RSV-PolyI:C groups when compared with the other groups, but no significant difference was noted between these two groups (Figure 6A). After infection with hMPV or RSV, the levels of TNF-α were markedly increased compared with the levels in uninfected cells, and the increase in the RSV group was larger than that in the hMPV group (*P*<0.05). The levels of TNF-α were higher in the RSV-PolyI:C and hMPV-PolyI:C groups compared with the respective virus alone groups; however, the TNF-α levels were much higher in the RSV-PolyI:C group than that all other groups (Figure 6B). To study whether the expression of other inflammatory cytokines as the same asTNF-α, we also measured the levels of IL-8 which also play a pivotal role in airway immune response to RSV infection [32]. The levels of IL-8 were markedly increased in hMPV, RSV, hMPV-PolyI:C and RSV-PolyI:C groups compared with the levels in uninfected cells, but there was no significant difference between hMPVand RSV (Figure 6C).

## Discussion

The severity of pulmonary inflammation in viral disease is determined by a combination of virus-induced damage and the host’s immune responses [33]. Currently, increasing evidence indicates that, in the pathogenesis of viral infection, host immune responses against pathogens play a larger role in determining the magnitude of inflammation and disease severity than the cytopathic effect induced by virus itself. Thus, the present study investigated the correlations between RSV and hMPV infections and TLR levels to explore the immunologic mechanisms underlying infections with these viruses and any potential differences in the mechanism between the two infections.

After successfully establishing in vitro and in vivo models for RSV and hMPV infections, we assessed the growth kinetics of both viruses. We found that the replication levels of RSV were slightly higher than those of hMPV when the input MOIs were the same (less that 1 log at various intervals). Additionally, as demonstrated by the analysis of lung histopathology, pulmonary lesions of RSV-infected mice were more severe than those in hMPV-infected mice. Because it has been previously demonstrated that RSV grew more efficiently in various cell cultures and animal models, it was not surprising that RSV reached a higher titer as compared with hMPV both in vitro and in mouse lungs. Whether the more severe pulmonary pathology we observed was caused solely by higher viral load remains unanswered.

We have demonstrated that A549 cells express TLR1-10 at similar levels as other respiratory tract cell strains [34,35,36]. In the present study, the levels of TLRs did not increase significantly in A549 cells exposed to UV-hMPV or UV–RSV, which is consistent with a previous study [35] and suggests that the increased levels of TLRs are dependent on viral replication. Furthermore, our results demonstrated that the levels of most TLRs were significantly up-regulated after hMPV or RSV infection when compared with uninfected A549 cells or cells treated with UV-inactivated viruses. The results suggest that this response is not pathogen-specific and are general features of anti-viral immune responses. The results from the real-time quantitative PCR measurements indicated that the levels of TLR3-4 and TLT7-8 increased in a time-dependent manner after RSV infection, and these levels continued to increase up to 24 h after infection. Of all TLRs, TLR3 was the most significantly increased which was consistent with other studies [37,38]. Similarly, after hMPV infection, the levels of TLR3-4 and TLT7-9 increased in a time-dependent manner; however the levels of TLR3-4 and TLR7-9 peaked at 9 and 12 h after infection, respectively, and then began to decrease. Overall, the levels of TLR3 induced by hMPV infection were significantly lower than those after RSV infection.

The results of the *in vitro* experiments revealed that the levels of the TLRs increased to varying extents after RSV or hMPV infections, and we further investigated these changes in the lungs of BALB/c mice after hMPV or RSV infection *in vivo*. These *in vivo* studies indicated that, after viral infection, the levels of TLR3-4 and especially TLR7-8 were increased in the lungs. Brian et al [33] have also reported increased levels of TLR3, TLR7 and TLR9 following RSV infection of mice. Matthew et al [39] found that TLR2 is critical for an efficient immune response against RSV infection, suggesting that an antiviral immune response can be initiated after activation of TLR pathways by RSV and hMPV infections *in vivo*. Previous studies have demonstrated that TLR7/8 can recognize viral ssRNA and activate immune responses [34,40,41]. In the present study, we found that TLR7-8 increased a greater amount than TLR3-4 after hMPV infection and also than TLR4 after RSV infection, which was differ from the *in vitro* experiments. Whether TLR7-8 pathway play an more important role in mice than in human body was still unclear. Furthermore, in both the *in vitro* and *in vivo* experiments, the TLR3 levels were significantly increased after RSV infection compared with hMPV infection.

Our analysis of TLR3 protein expression indicated that A549 cells primarily contain intracellular TLR3. Poly I:C stimulation can increase both intracellular and surface TLR3 protein expression, but the protein was still largely intracellular, RSV and hMPV infection also increased the intracellular TLR3 protein levels above those in uninfected cells. However, both the mRNA and protein levels of TLR3 were significantly higher after RSV infection than after hMPV infection, which suggests that TLR3 might play an important role in RSV infection. The specific ligand of TLR3 is double-stranded RNA (dsRNA), which can be produced in a great quantities when RSV is replicated and transcribed [37]. Thus, it is possible that the increased levels of TLR3 observed after RSV infection indicate that more dsRNA is produced upon RSV replication compared with hMPV replication.

Previous studies have suggested that viral nucleic acids can be recognized by TLR3, and TLR7-9 in cells after viral infection, which would then lead to the production of type I interferon and inflammatory cytokines via the IRF-3 and Nf-κB pathways. Additionally, the glycoproteins of some viruses can be recognized by TLRs located in the cell membrane, especially the TLR4, which induces expressions of type I interferon and inflammatory cytokines via TRIF and TRAM pathways [31]. Our results indicate that 24 h after RSV infection, the levels of TLR3-4 and TLR7-8 were higher than those after hMPV infection, especially TLR3. Thus, we hypothesized that the levels of IFN-α and inflammatory cytokines would be higher after infection with RSV than infection with hMPV.

To confirm this hypothesis, the levels of IFN-α, TNF-α and IL-8 were measured after infection with hMPV or RSV. The results indicated that, 24 h after infection, TNF-α production was significantly higher in cells infected with either RSV or RSV-PolyI:C than in cells treated with either hMPV alone or hMPV-PolyI:C. However, the production of IL-8 was no significant difference between hMPVand RSV or between hMPV-PolyI:C and RSV-PolyI:C. These findings indicate that RSV is able to induce more TNF-α production, which is consistent with the observed up-regulation of TLRs upon infection. TNF-α is an important pro-inflammatory factor that plays a critical role in the formation and development of pulmonary inflammatory lesions induced by viral infection. RSV can induce more severe pulmonary inflammation than hMPV, that TNF-α may have a important role in this disease progression.

Poly I:C is a synthetic analog of double-stranded RNA, the ligand of TLR3, and thus can stimulate the TLR3 pathway [42,43,44]. TLR3 and TNF-α are significantly increased after hMPV-PolyI:C treatment compared with hMPV alone, indicating that the activation of the TLR3 pathway plays an important role in the production of TNF-α.

We found that the IFN-α levels increased upon infection when compared with uninfected cells. However, it was inconsistent with the upregulation of TLRs observed upon infection, the IFN-α levels in RSV group or RSV-PolyI:C groups were not higher than that in hMPV group or hMPV-PolyI:C group. IFN-α is an important cytokines in anti-viral immune response, which can limit viral replication until virus-specific host defense mechanisms develop in many respiratory virus infections. Antonieta et al [45,46] found that the ability of hMPV to directly induce IFN-α production was stronger than that of RSV in mouse models, and it is more responsive to IFN-α. Some research suggested the RSV nonstructural proteins NS1 and NS2 have been implicated in virus-induced inhibition of TNF-α/β [47,48]. Our findings also suggested that hMPV itself may promote IFN-α production, and this effect may be due to the lack of two nonstructural proteins (NS1 and NS2) in hMPV. But unlike Antonieta’s finding, our results showed IFN-α levels were no difference between RSV and hMPV, this may be due to our results from the A549 cell line, while their results from mice.

In summary, our results suggest that, after hMPV or RSV infection, the levels of TLRs are up-regulated *in vitro* and *in vivo*, and *in vitro* this increase in TLR levels is related to viral replication. The role of TLR7-8 pathway in the anti-viral immune response in mice, may be different from which in human infection. RSV infection induces more TLR3 expression and TNFα production than hMPV does, which may partially explain the more severe inflammation observed after RSV infection compared with hMPV infection.

## References

[1] Hall CB (2001) Respiratory syncytiaI virus and parainfluenza virus. N EngJ Med 344(25): 1917-1928. doi:10.1056/NEJM200106213442507. 10.1056/NEJM200106213442507 11419430

[2] Sigurs N, Bjarnason R, Sigurbergsson F, Kjellman B (2000) Respiratory syneytial virus bronchiolitis in infancy is an important risk factor for asthma and allergy at age 7. Am J Respir Crit Care Med 161(5): 150l-1507. 10.1164/ajrccm.161.5.9906076 10806145

[3] Van den Hoogen BG, De Jong JC, Groen J, Kuiken T, de Groot R et al. (2001) A newly discovered human pneumovirus isolated from young children with respiratory tract disease. Nat Med 7(6): 719-724. doi:10.1038/89098. PubMed: 11385510. 11385510 10.1038/89098 PMC7095854

[4] Boivin G, Abed Y, Pelletier G, Ruel L, Moisan D et al. (2002) Virological features and clinical manifestations associated with human metapneumovirus: a new paramyxovirus responsible for acute respiratory-tract infections in all age groups. J Infect Dis 186(9): 1330-1334. doi:10.1086/344319. PubMed: 12402203. 12402203 10.1086/344319

[5] van den Hoogen BG, Bestebroer TM, Osterhaus AD, Fouchier RA (2002) Analysis of the genomic sequence of a human metapneumovirus. Virology 295: 119-132. doi:10.1006/viro.2001.1355. PubMed: 12033771. 12033771 10.1006/viro.2001.1355

[6] Williams JV, Harris PA, Tollefson SJ, Halburnt-Rush LL, Pingsterhaus JM et al. (2004) Human metapneumovirus and lower respiratory tract disease in otherwise healthy infants and children. N Engl J Med 350(5): 443-450. doi:10.1056/NEJMoa025472. PubMed: 14749452. 14749452 10.1056/NEJMoa025472 PMC1831873

[7] Falsey AR, Erdman D, Anderson LJ, Walsh EE (2003) Human metapneumovirus infections in young and elderly adults. J Infect Dis 187(5): 785-790. doi:10.1086/367901. PubMed: 12599052. 12599052 10.1086/367901

[8] Von Linstow ML, Larsen HH, Eugen-Olsen J, Koch A (2004) Human Metapneumovirus and Respiratory Syncytial Virus in Hospitalized Danish Children with Acute Respiratory Tract Infection. Scand J Infect Dis 36: 578-584. doi:10.1080/00365540410018166. PubMed: 15370669. 15370669 10.1080/00365540410018166

[9] Viazov S, Ratjen F, Scheidhauer R, Fiedler M, Roggendorf M (2003) High prevalence of human metapneumovirus infection in young children and genetic heterogeneity of the viral isolates. J Clin Microbiol 41(7): 3043-3045. doi:10.1128/JCM.41.7.3043-3045.2003. PubMed: 12843040. 12843040 10.1128/JCM.41.7.3043-3045.2003 PMC165336

[10] Marguet C, Lubrano M, Gueudin M, Le Roux P (2009) In very young infants severity of acute bronchiolitis depends on carried viruses. PLOS ONE 4(2): e4596. doi:10.1371/journal.pone.0004596. PubMed: 19240806. 19240806 10.1371/journal.pone.0004596 PMC2644758

[11] Akira S, Takeda K, Kaisho T (2001) Toll-like receptors: critical proteins linking innate and acquired immunity. Nat Immunol 2: 675-680. doi:10.1038/90609. PubMed: 11477402. 11477402 10.1038/90609

[12] Akira S (2001) Toll-like receptors and innate immunity. Adv Immunol 78: 1-56. doi:10.1016/S0065-2776(01)78001-7. PubMed: 11432202. 11432202 10.1016/s0065-2776(01)78001-7

[13] Haynes LM, Moore DD, Kurt-Jones EA, Finberg RW, Anderson LJ et al. (2001) Involvement of Toll-like receptor 4 in innate immunity to respiratory syncytial virus. J Virol 75(22): 10730-10737. doi:10.1128/JVI.75.22.10730-10737.2001. PubMed: 11602714. 11602714 10.1128/JVI.75.22.10730-10737.2001 PMC114654

[14] Alexopoulou L, Holt AC, Medzhitov R, Flavell RA (2001) Recognition of doublestranded RNA and activation of NF-kappaB by Toll-like receptor 3. Nature 413(6857): 732-738. doi:10.1038/35099560. PubMed: 11607032. 11607032 10.1038/35099560

[15] Hemmi H, Kaisho T, Takeuchi O, Sato S, Sanjo H et al. (2002) Small anti-viral compounds activate immune cells via the TLR7 MyD88-dependent signaling pathway. Nat Immunol 3(2): 196-200. doi:10.1038/ni758. PubMed: 11812998. 11812998 10.1038/ni758

[16] Baron S, Singh I, Chopra A, Coppenhaver D, Pan J (2000) Innate antiviral defenses in body fluids and tissues. Antiviral Res 48: 71–89. doi:10.1016/S0166-3542(00)00126-1. PubMed: 11114410. 11114410 10.1016/S0166-3542(00)00126-1 PMC7125796

[17] Le Bon A, Tough DF (2002) Links between innate and adaptive immunity via type I interferon. Curr Opin Immunol 14: 432–436. doi:10.1016/S0952-7915(02)00354-0. PubMed: 12088676. 12088676 10.1016/s0952-7915(02)00354-0

[18] Janssen R, Bont L, Siezen CL, Hodemaekers HM, Ermers MJ et al. (2007) Genetic susceptibility to respiratory syncytial virus bronchiolitis is predominantly associated with innate immune genes. Infect Dis 196(6): 826-834. doi:10.1086/520886. PubMed: 17703412. 10.1086/520886 17703412

[19] Noah TL, Ivins SS, Murphy P, Kazachkova I, Moats-Staats B et al. (2002) Chemokines and inflammation in the nasal passages of infants with respiratory syncytial virus bronchiolitis. Clin Immunol 104: 86-95. doi:10.1006/clim.2002.5248. PubMed: 12139952. 12139952 10.1006/clim.2002.5248

[20] Karin M, Ben-Neriah Y (2000) Phosphorylation meets ubiquitination: the control of NF-κB activity. Annu Rev Immunol 18(5): 621-663. 10837071 10.1146/annurev.immunol.18.1.621

[21] Sheeran P, Jafri H, Carubelli C, Saavedra J, Johnson C et al. (1999) Elevated cytokine concentrations in the nasopharyngeal and tracheal secretions of children with respiratory syncytial virus disease. Pediatr Infect Dis J 18(2): 115-122. doi:10.1097/00006454-199902000-00007. PubMed: 10048682. 10048682 10.1097/00006454-199902000-00007

[22] Abraham E (2003) Neutrophils and acute lung injury. Crit Care Med 31(Suppl 4): S195-S199. doi:10.1097/00003246-200301000-00030. PubMed: 12682440. 12682440 10.1097/01.CCM.0000057843.47705.E8

[23] Zhao MQ, Stoler MH, Liu AN, Wei B, Soguero C et al. (2000) Alveolar epithelial cell chemokine expression triggered by antigen-specific cytolytic T cell recognition. J Clin Invest 106(6): R49-R45. doi:10.1172/JCI9786. PubMed: 10995793. 10995793 10.1172/JCI9786 PMC381394

[24] Holtzman MJ, Shornick LP, Grayson MH, Kim EY, Tyner JW et al. (2004) “Hit-and-run” effects of paramyxoviruses as a basis for chronic airway disease. Pediatr Infect Dis J 23(11): S235-S245. PubMed: 15577579. 15577579 10.1097/01.inf.0000144674.24802.c1

[25] Olszewska-Pazdrak B, Casola A, Saito T, Alam R, Crowe SE et al. (1998) Cell-specific expression of RANTES, MCP-1, and MIP-1alpha by lower airway epithelial cells and eosinophils infected with respiratory syncytial virus. J Virol 72(6): 4756-4764. PubMed: 9573240. 9573240 10.1128/jvi.72.6.4756-4764.1998 PMC110009

[26] Ueba O (1978) Respiratory syncytial virus. I. concentration and purification of the infectious virus. Acta Med Okayama 32(4): 265-272. PubMed: 153087. 153087

[27] Garofalo RP, Mei F, Espejo R, Ye G, Haeberle H et al. (1996) Respiratory syncytial virus infection of human respiratory epithelial cells upregulates class I MHC expression through the induction of IFN-β and IL-1α. J Immunol 157: 2506-2513. PubMed: 8805651. 8805651

[28] Sha Q, Truong-Tran AQ, Plitt JR, Beck LA, Schleimer RP (2004) Activation of airway epithelial cells by toll-like receptor agonists. Am J Respir Cell Mol Biol 31: 358-364. doi:10.1165/rcmb.2003-0388OC. PubMed: 15191912. 15191912 10.1165/rcmb.2003-0388OC

[29] Cimolai N, Taylor GP, Mah D, Morrison BJ (1992) Definition and application of a histopathological scoring scheme for an animal model of acute Mycoplasma pneumoniae pulmonary infection. Microbiol Immunol 36: 465-478. PubMed: 1513263. 1513263 10.1111/j.1348-0421.1992.tb02045.x

[30] Genestier L, Taillardet M, Mondiere P, Gheit H, Bella C et al. (2007) TLR Agonists Selectively Promote Terminal Plasma Cell Differentiation of B Cell Subsets Specialized in Thymus-Independent Responses. J Immunol 178(12): 7779-7786. PubMed: 17548615. 17548615 10.4049/jimmunol.178.12.7779

[31] Kawai T, Akira S (2006) TLR signaling. Cell Death Differ 13(5): 816-825. doi:10.1038/sj.cdd.4401850. PubMed: 16410796. 16410796 10.1038/sj.cdd.4401850

[32] Yoon JS, Kim HH, Lee Y, Lee JS (2007) Cytokine induction by respiratory syncytial virus and adenovirus in bronchial epithelial cells. Pediatr Pulmonol 42: 277–282. doi:10.1002/ppul.20574. PubMed: 17245736. 17245736 10.1002/ppul.20574

[33] Rudd BD, Smit JJ, Flavell RA, Alexopoulou L, Schaller MA et al. (2006) Deletion of TLR3 Alters the Pulmonary Immune Environment and Mucus Production during Respiratory Syncytial Virus Infection. J Immunol 176(3): 1937-1942. PubMed: 16424225. 16424225 10.4049/jimmunol.176.3.1937

[34] Heil F, Hemmi H, Hochrein H, Ampenberger F, Kirschning C et al. (2004) Species-specific recognition of single-stranded RNA via Toll-like receptor 7 and 8. Science 303(5663): 1526-1529. doi:10.1126/science.1093620. PubMed: 14976262. 14976262 10.1126/science.1093620

[35] Xie XH, Law HK, Wang LJ, Li X, Yang XQ et al. (2009) Lipopolysaccharide Induces IL-6 Production in Respiratory Syncytial Virus-Infected Airway Epithelial Cells Through the Toll-Like Receptor 4 Signaling Pathway. Pediatr Res 65(2): 156-162. doi:10.1203/PDR.0b013e318191f5c6. PubMed: 18948841. 18948841 10.1203/PDR.0b013e318191f5c6

[36] Sha Q, Truong-Tran AQ, Plitt JR, Beck LA, Schleimer RP (2004) Activation of Airway Epithelial Cells by Toll-Like Receptor Agonists. Am J Respir Cell Mol Biol 31(3): 358-364. doi:10.1165/rcmb.2003-0388OC. PubMed: 15191912. 15191912 10.1165/rcmb.2003-0388OC

[37] Groskreutz DJ, Monick MM, Powers LS, Yarovinsky TO, Look DC et al. (2006) Respiratory Syncytial Virus Induces TLR3 Protein and Protein Kinase R, Leading to Increased Double-Stranded RNA Responsiveness in Airway Epithelial Cells. J Immunol 176(3): 1733-1740. PubMed: 16424203. 16424203 10.4049/jimmunol.176.3.1733

[38] Monick MM, Yarovinsky TO, Powers LS, Butler NS, Carter AB et al. (2003) Respiratory Syncytial Virus Up-regulates TLR4 and Sensitizes Airway Epithelial Cells to Endotoxin. J Biol Chem 278(52): 53035-53044. doi:10.1074/jbc.M308093200. PubMed: 14565959. 14565959 10.1074/jbc.M308093200

[39] Murawski MR, Bowen GN, Cerny AM, Anderson LJ, Haynes LM et al. (2009) Respiratory Syncytial Virus Activates Innate Immunity through Toll-Like Receptor 2. J Virol 83(3): 1492-1500. doi:10.1128/JVI.00671-08. PubMed: 19019963. 19019963 10.1128/JVI.00671-08 PMC2620898

[40] Diebold SS, Kaisho T, Hemmi H, Akira S, Reis e Sousa C (2004) Innate antiviral responses by means of TLR7-mediated recognition of single-stranded RNA. Science 303(5663): 1529-1531. doi:10.1126/science.1093616. PubMed: 14976261. 14976261 10.1126/science.1093616

[41] Lund JM, Alexopoulou L, Sato A, Karow M, Adams NC et al. (2004) Recognition of single-stranded RNA viruses by Toll-like receptor 7. Proc Natl Acad Sci U S A 101(15): 5598-5603. doi:10.1073/pnas.0400937101. PubMed: 15034168. 15034168 10.1073/pnas.0400937101 PMC397437

[42] Gern JE, French DA, Grindle KA, Brockman-Schneider RA, Konno S et al. (2003) Double-stranded RNA induces the synthesis of specific chemokines by bronchial epithelial cells. Am J Respir Cell Mol Biol 28: 731-737. doi:10.1165/rcmb.2002-0055OC. PubMed: 12600836. 12600836 10.1165/rcmb.2002-0055OC

[43] Guillot L, Le Goffic R, Bloch S, Escriou N, Akira S et al. (2005) Involvement of toll-like receptor 3 in the immune response of lung epithelial cells to double-stranded RNA and influenza a virus. J Biol Chem 280: 5571-5580. PubMed: 15579900. 15579900 10.1074/jbc.M410592200

[44] Matsukura S, Kokubu F, Kurokawa M, Kawaguchi M, Ieki K et al. (2006) Synthetic doublestranded RNA induces multiple genes related to inflammation through toll-like receptor 3 depending on NF-kB and/or IRF-3 in airway epithelial cells. Clin Exp Allergy 36: 1049-1062. doi:10.1111/j.1365-2222.2006.02530.x. PubMed: 16911361. 16911361 10.1111/j.1365-2222.2006.02530.x

[45] Guerrero-Plata A, Baron S, Poast JS, Adegboyega PA, Casola A et al. (2005) Activity and Regulation of Alpha Interferon in Respiratory Syncytial Virus and Human Metapneumovirus Experimental Infections. J Virol 79(16): 10190-10199. doi:10.1128/JVI.79.16.10190-10199.2005. PubMed: 16051812. 16051812 10.1128/JVI.79.16.10190-10199.2005 PMC1182647

[46] Guerrero-Plata A, Casola A, Garofalo RP (2005) Human Metapneumovirus Induces a Profile of Lung Cytokines Distinct from That of Respiratory Syncytial Virus. J Virol 79(23): 14992-14997. doi:10.1128/JVI.79.23.14992-14997.2005. PubMed: 16282501. 16282501 10.1128/JVI.79.23.14992-14997.2005 PMC1287587

[47] Spann KM, Tran KC, Chi B, Rabin RL, Collins PL (2004) Suppression of the induction of alpha, beta, and lambda interferons by the NS1 and NS2 proteins of human respiratory syncytial virus in human epithelial cells and macrophages [corrected]. J Virol 78: 4363-4369. doi:10.1128/JVI.78.8.4363-4369.2004. PubMed: 15047850. 15047850 10.1128/JVI.78.8.4363-4369.2004 PMC374276

[48] Valarcher JF, Furze J, Wyld S, Cook R, Conzelmann KK et al. (2003) Role of alpha/beta interferons in the attenuation and immunogenicity of recombinant bovine respiratory syncytial viruses lacking NS proteins. J Virol 77: 8426-8439. doi:10.1128/JVI.77.15.8426-8439.2003. PubMed: 12857912. 12857912 10.1128/JVI.77.15.8426-8439.2003 PMC165239

